# Assessing community readiness online: a concurrent validation study

**DOI:** 10.1186/s12889-015-1953-5

**Published:** 2015-07-02

**Authors:** Iordan Kostadinov, Mark Daniel, Linda Stanley, Margaret Cargo

**Affiliations:** School of Population Health, University of South Australia, Adelaide, Australia; Department of Medicine, St. Vincent’s Hospital, The University of Melbourne, Fitzroy, Australia; South Australian Health and Medical Research Institute, Adelaide, Australia; College of Natural Sciences, Tri-Ethnic Center, Colorado State University, Fort Collins, USA

**Keywords:** Community readiness, Concurrent validation, Community-based interventions, Obesity prevention

## Abstract

**Background:**

Community readiness for facilitation and uptake of interventions can impact the success of community-based prevention efforts. As currently practiced, measuring community readiness can be a resource intensive process, compromising its use in evaluating multisite community-based prevention efforts. The purpose of this study was to develop, test and validate a more efficient online version of an existing community readiness tool and identify potential problems in completing assessments. This study was conducted in the context of a complex community-based childhood obesity prevention program in South Australia.

**Methods:**

Following pre-testing, an online version of the community readiness tool was created, wherein respondents, with detailed knowledge of their community and prevention efforts, rated their communities on five anchored rating scales (Knowledge of Efforts, Leadership, Knowledge of the Issue, Community Climate, and Resources). Respondents completed the standard, over-the-phone community readiness interview (“gold standard”) and the new online survey. Paired *t*-test, St. Laurent’s correlation coefficient and intra-class correlation (ICC) were used to determine the validity of the online tool. Contact summary forms were completed after each interview to capture interview quality.

**Results:**

Twenty-five respondents completed both assessments. There was a statistically significant difference in the overall community readiness scores between the two methods (paired *t*-test *p* = 0.03); online scores were consistently higher than interview scores. St. Laurent’s correlation of 0.58 (95 % CI 0.42–0.73) was moderate; the ICC of 0.65 (95 % CI 0.35–0.83) was good. Only for the leadership and resources dimensions was there no statistically significant difference between the scores from the two methods (*p* = 0.61, *p* = 0.08 respectively). St Laurent’s correlation (r = 0.83, 95 % CI 0.71–0.92) and the ICC (0.78, 95 % CI 0.57–0.90) were excellent for leadership. Qualitative results from the standard interview method suggest that some respondents felt reluctant to answer questions on behalf of other community members. This may have impacted their self-selected ratings and/or responses to questions during the interview.

**Conclusions:**

Concurrent validity for the online method was supported for the Leadership dimension only. However, the online method holds promise as it reduces time and resource burden, allowing for a quicker return of results to the community to inform program planning, implementation and evaluations to improve community health.

## Background

The implementation and ultimate success of community-based health interventions may be greatly impacted by contextual factors such as a community’s social and built environments [1–3]. A comprehensive evaluation of the outcomes of prevention efforts requires understanding the contextual factors that may have impacted these outcomes [4].

The level of effort that a community is prepared to apply and ready to respond to a particular issue is known as ‘community readiness to change’. It combines several contextual constructs which can be difficult to quantify yet are key to understanding the success or lack thereof of community-based health efforts [5, 6]. Contextual constructs often cited as key dimensions of community readiness include community awareness and knowledge of the issue and current efforts to address it, leadership knowledge and support, and community resources and capacity [7, 8].

The Community Readiness Tool (CRT) developed by Edwards et al. [5] is widely used for assessing community readiness and has been identified as useful for evaluation and program planning purposes [9–12]. This tool was created to apply the Community Readiness Model (CRM) which combines and expands upon the personal stages of change [13] and community development principles [14]. The CRM defines five dimensions of community readiness which are scored through the CRT. These dimensions are: Community Knowledge of Existing Efforts, Leadership, Community Climate, Community Knowledge about the Issue, and Resources. The standard method of administering the CRT is detailed elsewhere [15]. Briefly, in order to assess community readiness the CRT protocol requires 4–6 semi-structured interviews per community, each taking approximately 45 minutes. Interviews are transcribed and scored by two independent scorers using anchored rating scales. The CRT has been used successfully in a number of different settings [16–19] for a wide variety of applications. However, this process is time and resource intensive, limiting use of CRT to small-scale or single site communities, and preventing community readiness evaluation of large scale, multi-site, community-based health promotion initiatives [20, 21].

To improve the resource efficiency of the CRT, we developed an online survey version of the tool. This report describes our concurrent validation of the online survey using the standard phone version of the tool against our online protocol. We hypothesised that there would be no difference in the overall community readiness score between the gold standard interview method and the online survey version. We also hypothesised no differences in dimension scores between the two methods.

## Methods

### Study setting

The setting for this study is the Obesity Prevention and Lifestyle (OPAL) program currently underway in 21 rural and urban communities in South Australia (*n* = 20) and the Northern Territory (*n* = 1) [22]. The goal of OPAL is “to improve eating and activity patterns of children, through families and communities in OPAL regions, and thereby increase the proportion of 0–18 year olds in the healthy weight range”. Each participating community received $75,000 per year in funding for project implementation as well as two full time staff. Interventions are implemented within each community at the discretion of the local staff who consult with host agencies, partners and community members, although they must align with the OPAL program framework and principles drawn from the French EPODE model [23]. The 21 communities have staggered starting points over four years: the first set of communities began in late 2009 and the final stage commenced in late 2012. The communities have varying demographic and geographic profiles and contain between 1 and 45 suburbs (mean = 10.3, median = 9). For this study, the suburb was the unit of analysis. Each community provided at least one key informant who completed the CRT for a chosen suburb within the community. A full community readiness assessment was not completed for any given suburb or community as this study focussed on the validation of the online version of the CRT; the CRT was validated at the individual level, not at the community level. In other words, we are comparing whether the scores for the gold standard interview for an individual were the same as the individual’s online scores.

### Ethical approval and consent

Ethical approval for this study was granted by the South Australian Department of Health Human Research Ethics Committee (reference number 327/11/2012) and the University of South Australia Human Research Ethics Committee (reference number 25002). All participants were given an information sheet, had the opportunity to ask questions about the study, and signed a consent form prior to participating.

### Sampling and validation

Key informants (*n* = 30) for the concurrent validation study were selected based on their knowledge of obesity prevention activities being implemented within a given suburb. The positions of the key informants varied between suburbs and included elected councillors, local council staff, teachers, childcare centre and other non-government organisation directors, and OPAL staff. Key informants were asked to complete two versions of the CRT. Firstly they were invited to participate in the gold standard, telephone interview. This semi-structured interview consisted of the 20 core questions of the CRT with the required adaptations to refer to the issue at hand (childhood obesity prevention) and the community (in this case suburb) in question. In order to ensure the best chance at validity, the interviewer underwent rigorous training by experienced CRT researchers on interviewing best practices. Immediately following the telephone interview a contact summary form was completed by the interviewer, as recommended by Miles, Huberman and Saldana [24]. It provided opportunity for the interviewer to comment on the overall quality of the interview (i.e., depth of responses, any interruptions to the interview, tentativeness in responding to questions), sections of the interview which were problematic or poorly answered (i.e. respondent did not possess requisite information), or any other noteworthy comments (i.e. very low levels of readiness, buy-in from the respondent). A minimum of four weeks later, participants were invited to complete the online survey version of the tool. This ‘wash out’ period was to ensure that the phone interview questions did not influence the online responses. Informants completed the phone interview first because exposing informants to the anchored rating scales prior to the phone interview could have compromised the gold standard methodology. Change in readiness over the one month ‘wash out’ period was unlikely given the difficulty in increasing or decreasing community readiness. Previous research using the CRT over multiple time points has found that every year of intervention was associated with a 0.6 increase in overall community readiness [25].

To promote high quality scoring, interviews were transcribed and scored by two expert scorers who independently read through each interview transcript six times – once without scoring and then five more times to score each dimension separately. The dimensions were scored on anchored rating scales with scorers starting at the lowest readiness level and looking for evidence of the one above until no such evidence could be found. Once the two scorers completed their independent scoring, they came together to discuss their scores and come to a final consensus score for each dimension. The overall community readiness score (between 1 and 9) was then calculated by taking the average of each dimension score [15].

### Online version of the CRT

Development of the online version of the tool was facilitated using Survey Monkey online survey software. The survey began with the same definition of the issue (childhood obesity) as the standard interview. However, unlike the gold standard interview version of the CRT where respondents are asked questions with their responses being scored on the anchored rating scales, the online version of the tool asks respondents to directly score a specific suburb of interest on each of five anchored ranking scales corresponding to the five community readiness dimensions (Community Knowledge of Efforts, Leadership, Community Knowledge of the Issue, Community Climate, and Resources). The anchored rating scales can be found in the Community Readiness Handbook [15]. Thus, the scoring process is conducted by the respondent online, rather than by the researcher through a transcript. The core questions of the gold standard tool are used as prompts in the instructions for each dimension, but are not specifically answered in the online tool. Once the respondent had scored each of the five anchored rating scales an overall community readiness score (between 1 and 9) was calculated by taking the average of each dimension score.

The online survey underwent a pre-testing process whereby three key informants completed the online survey and provided formal feedback through a semi-structured recorded phone interview. This feedback was used to improve the online version of the tool, with alterations made to survey instructions and visual elements of the anchored rating scales.

### Analysis

Scores on the gold standard (interview) and the online survey version of the tool were contrasted using St. Laurent’s gold standard correlation coefficient to assess the validity of the new administration method [26]. This test is used to compare two different methods of measurement when one is a gold standard. In addition, the intra-class correlation coefficient (two way model with fixed raters – ICC 3,1), paired *t*-test and Wilcoxon signed ranks test were used to further test the differences in scores between the two administration methods. The use of multiple statistical tests is recommended to overcome the shortcomings of any single procedure [27]. The Wilcoxon signed rank test was included as a non-parametric test in case normality assumptions were not met. Statistical tests were undertaken using the Pairs Module of WinPEPI v.11.39. *A priori* power calculation for a paired *t*-test estimated that 25 respondents were required to detect a mean difference of 0.50 in community readiness scores, with power of 0.80 and alpha of 0.05. Based on previous studies, the standard deviation was estimated at 0.85 [28]. Contact summary forms were analysed through qualitative content analysis by the first and last authors. The specific form of directed content analysis was used [29]. Initial coding categories of Interview Quality, Completeness of Information, and Salient/ Illuminating Issues were identified at the outset. Information within each category was coded to depict the range of responses or issues raised.

## Results

Thirty phone interviews were conducted with an average length of 33 min (range = 22–52, median = 32). However, two participants did not provide sufficient information during the interview to allow for scoring and three participants did not complete the online survey, leaving a final sample of 25 key informants. Average completion time of the online survey was 29 minutes (range = 10–60, median = 30).

The results for the overall and dimension scores are shown in Table 1. The online survey scored 0.39 (SD = 0.83) points higher than that for the phone interview method, with a range of −1.50 to 1.75 for difference scores. Overall community readiness scores for the telephone interview method ranged from 3.88 to 6.00 and the online scores ranged from 2.46 to 7.17. In the majority of cases (*n* = 18), the telephone and online scores were within 1 point of each other. The paired *t*-test revealed a statistically significant difference in overall scores between the two methods (*p* = 0.03). St. Laurent’s correlation coefficient was 0.58 (95 % CI 0.42–0.73), indicating a moderate correlation between the overall community readiness scores. The intra-class correlation calculated using a two-way model with fixed raters was 0.65 (95 % CI 0.35–0.83) a figure regarded as good reliability but less than the threshold for excellent (0.75) [30].

**Table 1:** Range of online and interview scores, average difference, St. Laurent’s Correlation coefficient, Intra-Class Correlation (ICC), Wilcoxon signed ranks test, and paired *t*-test for the interview (gold standard) and online survey versions of the CRT, dimension scores and overall CR score (*n* = 25)

Test	Community knowledge of existing efforts	Leadership	Community climate	Community knowledge of the issue	Resources	CR score
Range of online survey scores	3.00–6.50	1.75–7.75	1.00–6.75	2.00–7.75	1.00–7.75	2.46–7.17
Range of interview (gold standard) scores	2.75–6.25	1.25–7.50	2.00–5.25	1.50–6.25	6.00–7.50	3.88–6.00
Average difference^a^ (SD)	0.62 (1.07)	−0.12 (1.14)	0.92 (1.25)	1.21 (1.66)	−0.59 (1.61)	0.39 (0.83)
St. Laurent’s correlation (95 % CI)	0.51 (0.15,0.76)	0.83 (0.71,0.92)	0.58 (0.43,0.74)	0.50 (0.62–0.66)	0.66 (0.51–.0.80)	0.58 (0.42–0.73)
ICC (95 % CI)	0.56 (0.42,0.73)	0.78 (0.57,0.90)	0.61 (0.28,0.81)	0.367 (−0.03,0.67)	−0.36 (−.42,0.36)	0.65 (0.35,0.83)
Wilcoxon signed ranks test Z	−2.18	−0.46	−3.18	−2.83	−1.69	−2.38
Asymp. Sig. (2-tailed)	0.03	0.65	<0.00	0.01	0.09	0.02
Paired *t*-test *p*-value	0.01	0.61	<0.00^b^	0.002^b^	0.08^b^	0.03

^a^Online survey score minus interview (gold standard) score

^b^Variables not normally distributed

As data for three of the five (community climate, community knowledge of the issue, resources) dimension scores were not normally distributed, the Wilcoxon signed ranks test was used to test for differences between the two methods on these dimensions. Dimension scores for Leadership (paired *t*-test *p* = 0.61) and Resources (Wilcoxon signed ranks test, *p* = 0.09) did not differ between online and phone interview methods. Dimension scores for Knowledge of Existing Efforts (paired *t*-test, *p* = 0.01), Community Climate (Wilcoxon signed ranks test *p* < 0.00) and Community Knowledge about the Issue (Wilcoxon signed ranks test *p* = 0.01) were found to differ between administration methods. The data for the Resource dimension was highly skewed to scores above 6, consistent with the level of resourcing associated with the OPAL program. St. Laurent’s correlation coefficients were moderate (0.50–0.58) for all but the Leadership dimension, for which a strong correlation (0.83) was observed. Intra-class correlations demonstrated greater variation, but the Leadership dimension was the strongest at 0.78, indicating excellent reliability.

Content analysis of the telephone contact summary forms found that whilst most respondents provided answers with appropriate information for scoring, there was some unease when answering questions that provide information for scoring dimensions pertaining to the knowledge and attitudes of suburb residents (Table 2). Eleven respondents expressed reluctance to answer questions for the dimensions of Community Climate, Community Knowledge of the Issue, and Community Knowledge of the Existing Efforts as they did not feel they knew all of the residents and thus could not answer on their behalf. When this occurred, the interviewer clarified that the respondent was required only to answer for those whom they knew, resulting in elaborated answers in the majority of cases. This elaboration ensured that there was sufficient information for the scoring process to be completed. Questions relating to the dimensions of Leadership and Resources were answered without this same unease.

**Table 2:** Content analysis of contact summary form (*n* = 30)

Overall quality of interviews
a. Questions well answered, relevant information gathered (*n* = 19)
b. Most relevant information gathered, respondent may have been reluctant to answer some questions (*n* = 8)
c. Not all information there, may not be able to score (*n* = 3)
Completeness of information
a. All information gathered without trouble (*n* = 19)
b. Respondent was reluctant to give information on community climate and community knowledge of the issue (*n* = 7)
c. Respondent was reluctant to give information on community knowledge of existing efforts, community climate and community knowledge of the issue (*n* = 2)
d. Respondent was reluctant to give information on community climate (*n* = 2)
Salient, interesting, illuminating or important notes
a. No other salient, interesting, illuminating or important notes (*n* = 23)
b. Respondent did not want to use the term childhood obesity, instead referred to physical activity and healthy eating habits of children in the suburb (*n* = 4)
c. Low level of readiness in the suburb resulted in a shorter interview (*n* = 3)

## Discussion and conclusions

This concurrent validation study found that the overall results of an online administration of the CRT were significantly different from the gold standard interview method of administration, despite showing a good level of correlation across dimensions. Although the differences in scores between methods were not large, with most differences lying within one point, only the Leadership dimension demonstrated excellent reliability between the two methods of administration. Thus, only the Leadership dimension would appear to be ready for the online administration at this stage.

The largest differences between methods were observed for dimensions where respondents mentioned difficulty in answering for all residents of the suburb being rated – Community Knowledge of Efforts, Community Knowledge of the Issue, and Community Climate. Interestingly, for these dimensions, the interview scores were, on average, significantly lower than the online scores. This may reflect the lack of confidence felt by respondents in answering questions related to these dimensions, thus leading to less information for scorers to use in assigning a readiness level. Scorers are trained to use only the information provided by respondents and to only assign a level of readiness if wholly warranted by the respondents’ answers. Qualitative analyses uncovered weaknesses in both the online and telephone survey methods for these dimensions which had the largest quantitative differences. Dimensions relating to Community Knowledge of Existing Efforts, Community Climate and Knowledge of the Issue were at times answered poorly, with respondents reluctant to speculate on the attitudes of the broader community. Although the respondents are not required to know the attitudes and knowledge of everyone in their community, rather only those to whom they are exposed, the framing of the questions can be interpreted as though such knowledge is requisite. Improvements to the introductory explanation and core questions could help to alleviate these concerns. Despite the selection of informants based on their knowledge, organisation tenure within their place of work, and familiarity with their chosen suburb, some questions were difficult to answer. Respondents in this study required prompts to answer these questions fully; it is possible that more knowledgeable respondents would have provided richer answers for both the interview and online survey versions of the tool where such prompts are not possible.

Whilst previous research has identified time and resource challenges as limitations to the application of a full community readiness assessment [20, 21, 31], this tool has not undergone a formal qualitative component to unpack these difficulties. The results from the present study are the first in published literature to qualitatively assess interview quality of the CRT.

While improvements to the online survey are evidently still required, it is nonetheless considerably less difficult to administer than the standard telephone method. The online survey can be completed in the respondent’s own time, reducing burden not only for the evaluator, but for the participant. Furthermore, the online survey does not need to be recorded or transcribed before coding, and is simply scored by the participant themselves. The online method still requires participant recruitment, data entry and analysis as well as effort to set up and tailor the questions and scales to the appropriate issue and community. However, these tasks are also required in the standard telephone method. Whilst the online method does not remove all time and resource requirements, it does significantly lower them, making assessment of community readiness much more viable for large scale community-based programs where communities, neighbourhoods or suburbs are the unit of analysis.

All five community readiness dimensions are important for evaluating the current state of the community; however, assessing leadership readiness alone will provide valuable information for evaluators, community members and practitioners. Leaders within community may be formally elected representatives of the people or informal opinion leaders who have the capacity to influence their communities. Support from both formal [32] and informal [33] leaders is crucial to the success of public health programs. With the importance of leadership readiness data, and the statistical robustness of its online assessment put forward by the present study, the wider usage of the leadership component of the online community readiness survey is warranted.

Health program evaluations are increasingly incorporating greater flexibility and accountability, with results expected by the community on an on-going basis. This allows for greater responsiveness within interventions, but also necessitates simple and easy-to-use evaluation tools [34]. As a result, laborious and resource intensive instruments are making way for those which are straightforward and quick to administer [35]. This is particularly true in the current economic climate as time and resources for evaluation become increasingly scarce. Although the online medium is not without drawbacks, the ubiquitous nature of the online world in most developed countries holds great opportunities for health program evaluation. With further refinement, the online CRT will allow for simpler administration and completion, and consequently more accurate and speedy reporting. Ultimately, CRT may assist in informing and shaping prevention efforts in many areas of public health.

## Abbreviations

CRM: Community Readiness Model
CRT: Community Readiness Tool
ICC: Intra-Class Correlation
OPAL: Obesity Prevention and Lifestyle
